# Urban forest fragments as unexpected sanctuaries for the rare endemic ghost butterfly from the Atlantic forest

**DOI:** 10.1002/ece3.5596

**Published:** 2019-08-27

**Authors:** Antonio C. de Andrade, William Medeiros, Matthew Adams

**Affiliations:** ^1^ Departamento de Engenharia e Meio Ambiente Universidade Federal da Paraiba Rio Tinto Brazil; ^2^ Department of Geography Centre of Urban Enviroments University of Toronto Mississauga Toronto ON Canada; ^3^ Universidade Federal da Paraiba, PPGMA Rio Tinto Brazil; ^4^ Department of Geography University of Toronto Mississauga Toronto ON Canada

**Keywords:** agricultural practices, Allee effect, extinction, forest‐dependent species, insect communities, pollution, tropical landscape

## Abstract

Anthropogenic land expansion, particularly urbanization, is pervasive, dramatically modifies the environment and is a major threat to wildlife with its associated environmental stressors. Urban remnant vegetation can help mitigate these impacts and could be vital for species unable to survive in harsh urban environments. Although resembling nonurban habitats, urban vegetation remnants are subject to additional environmental stresses. Here, we evaluate the occurrence and density of the endemic ghost butterfly (*Morpho epistrophus nikolajewna*) that was once common, in the highly fragmented Atlantic forest of NE Brazil. We tested whether this butterfly would be found at lower densities in urban forest fragments of contrasting sizes as opposed to rural ones, given the number of environmental stressors found in urban areas. We surveyed 14 forest fragments (range 2.8 to over 3,000 ha) of semideciduous Atlantic forest in rural and urban locations using transect based distance sampling. The ghost butterflies showed strong seasonality; flying only from April to June. They were only identified in an urban fragment (515 ha), with an estimate of 720 individuals and a density 1.4 ind/ha. All forest fragments had experienced some level of logging in the past, which might have had an effect in the butterfly population. Nevertheless, rural forest fragments were subject to increased particulate matter concentrations, associated to biomass burning that we suggest might have had a more influential role driving the collapse of rural populations. Our findings show the importance of urban forest remnants to sustain population of this endangered species.

## Graphical abstract

A butterfly was once common in Atlantic forest of NE Brazil and now rare. The population collapse probably has been driven by smoke pollution, from agricultural practices, and aggravated by life‐history characteristic of this butterfly.

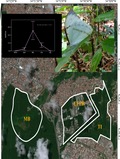

## 1 INTRODUCTION

In the 21st century, we are witnessing a dramatic expansion of anthropogenic land cover and uses, which include urbanization and large‐scale agriculture that have overall negative impacts on biodiversity and cause enormous environmental changes (Maxwell, Fuller, Brooks, & Watson, 2016; McDonald, Marcotullio, & Guneralp, 2013; Seto, Guneralp, & Hutyra, 2012; Tilman et al., 2001). Although land conversion to agriculture and urbanization both cause fragmentation and loss of natural habitats, some authors consider urbanization as a more extreme environmental change and as a major driver of global biodiversity loss (Aronson et al., 2014; McDonald, Kareiva, & Forman, 2008; McKinney, 2006; Seto et al., 2012). Indeed, urbanization is a complex process of physical changes that result in the removal and replacement of natural habitat with impermeable structures, the fragmentation and isolation of remaining habitats, a loss of biodiversity, and drastic change in species community composition (e.g., Aronson et al., 2014; Grimm et al., 2008). Although native habitat remnants within an urban matrix resemble nonurban wild habitats, they are often subject to profound additional environmental stresses (Miller & Hobbs, 2002), such as prey or competition with domestic/invasive species, noise, air, and light pollution (de Andrade, Franzini, & Mesquita, 2019; Birnie‐Gauvin, Peiman, Gallagher, de Bruijn, Cooke, 2016; Grimm et al., 2008; Grubisic, Grunsven, Kyba, Manfrin, & Hölker, 2018).

Some studies forecast a fourfold increase in urban land in existing biodiversity hotspots by 2030, with the largest increases expected in South America, leading to severe impacts on wildlife (Guneralp & Seto, 2013; McDonald et al., 2013). This expansion fragments the remaining patches of natural habitat and increases their isolation. Nevertheless, the urban patchwork of remnant vegetation, with its forests of various sizes and degrees of isolation, can mitigate the negative effects of urbanization and are regarded as vital for many organisms that are unable to survive in the more modified and hostile urban environment (de Andrade et al. in review; Soga, Yamaura, Koike, & Gaston, 2014). Moreover, native vegetation remnants also represent an important reservoir of local and regional biodiversity (Angold et al., 2006; Aronson et al., 2014; Ives et al., 2016). Much of what we know about the effects of urbanization is influenced by the large amount of data available on birds and mammals (e.g., Aronson et al., 2014; Gallo, Fidino, Lehrer, & Magle, 2017; Magle, Hunt, Vernon, & Crooks, 2012), the responses of which may not be representative of many other taxa. Arthropods, for instance, are still an understudied group in urban areas (Magle et al. 2012), and we still have gaps in our knowledge on how urbanization affects insects (Leather, 2018; Mata et al., 2017).

Butterflies are one of the most studied groups of insects and are frequently considered to be good and efficient ecological indicators (Brown & Freitas, 2000; Thomas, 2005, 2016). Dirzo et al. (2014) analyzed the wealth of data available for Lepidoptera and found a consistent and substantial decline in global abundance and diversity over 40‐year period, which they posit has been caused by agriculture and urbanization disturbances. These authors found that abundance is about twofold higher in undisturbed sites compared to disturbed sites. However, some studies suggest that certain groups of butterflies can maintain viable populations in small urban fragments (e.g., Brown & Freitas, 2002). Despite this, our knowledge of Neotropical butterfly ecology is scarce, which has potential implications for their conservation (Bonebrake, Ponisio, Boggs, & Ehrlich, 2010).

Cities are increasingly being recognized as important areas for biodiversity conservation and refuges for threatened species (Aronson et al., 2014; Ives et al. 2016; Luna, Romero‐Vidal, Hiraldo, & Tella, 2018). However, urban environments pose a series of detrimental factors for wildlife (e.g., McDonald et al., 2008) and it is essential to understand how wildlife copes in urban environments when compared to nonurban environments. Given what we know about the effects of urbanization, the number of stressors can be much higher in cities compared to nonurban environments, which may negatively affect the wildlife that reside in urban forest fragments. In an era of global urban expansion and rapid environmental change, understanding how urbanization could affect wildlife, particularly endangered species is critical for conservation. The Atlantic forest hotspot, in eastern Brazil, is forecasted to experience 160% increase in urban areas by 2030 (Guneralp & Seto, 2013; Seto et al., 2012). To address conservation, policy decisions and manage populations of rare species, we require data on how populations of different species could fare in urban forest remnants (e.g., Luna et al., 2018).

**Figure 1:**
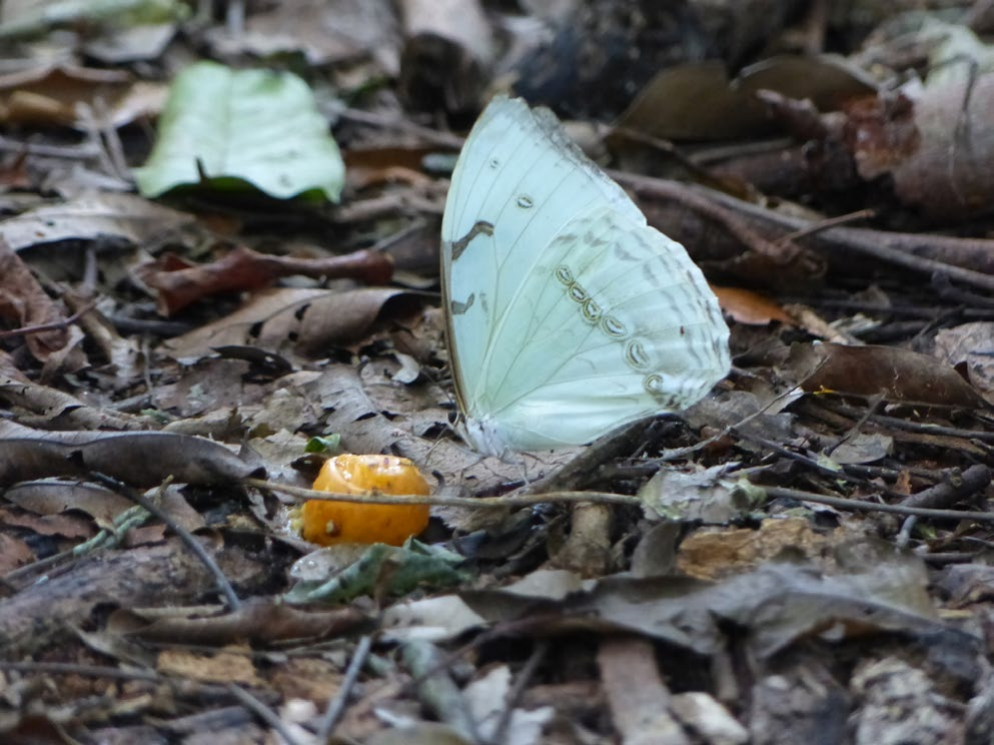
A ghost butterfly feeding on a fallen *Spondias mombin* fruit. This fruit has a length of about 4 cm

Here, we provide data on the occurrence and density of the rare ghost butterfly (*Morpho epistrophus nikolajewna*, Figure 1) in the highly fragmented Atlantic forest of NE Brazil. In the past this species was common, but with a restricted distribution occurring only in the coastal Atlantic forest of Alagoas, Paraiba, and Pernambuco (Freitas & Marini‐Filho, 2011). Their population seems to be dwindling, but the cause of the decline is yet unclear, although the loss, fragmentation and degradation of wild areas and use of pesticides are the most likely factors (Freitas & Marini‐Filho, 2011). The ghost butterfly is considered as critically endangered in the Brazilian list of threatened species (Freitas & Marini‐Filho, 2011), though information on population size is currently lacking. Here, we assess the occurrence of the ghost butterfly in forest fragments of contrasting sizes in urban and nonurban areas. We hypothesize that in urban forest fragments this butterfly will be found at lower density, due to a number of stressors, such as chemical and light pollution that have been demonstrated to negatively impact insects in the urban environment (Grimm et al., 2008; Grubisic et al., 2018; Hillstrom & Lindroth, 2008).

## 2 METHODS

### 2.1 Study areas

**Figure 2:**
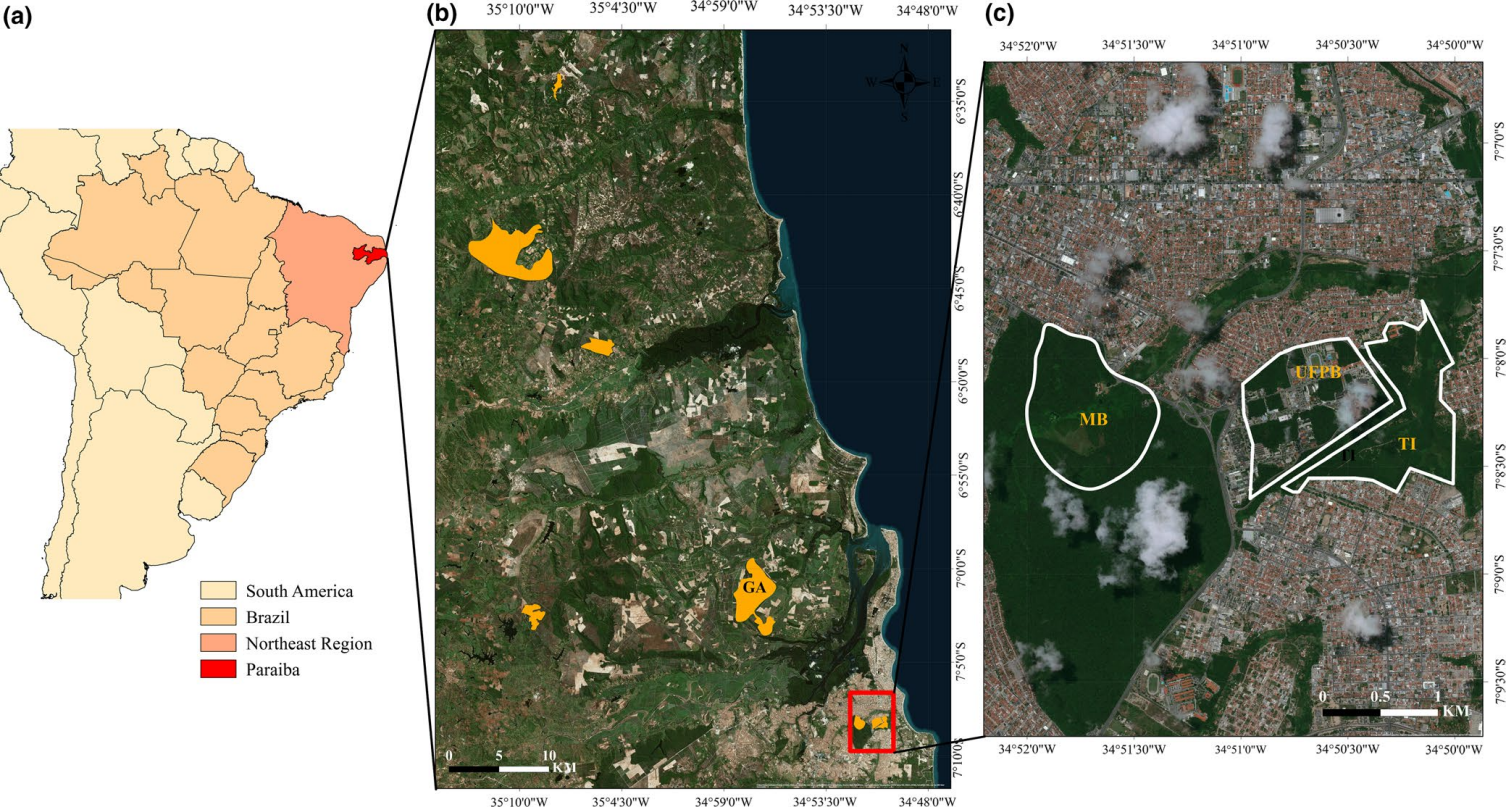
Location of the area (a) and forest fragments surveyed (b) and the urban fragments (c). UFPB (panel c) is the university campus, where the 7 largest fragments were surveyed. For details about the fragments see Table 1

We surveyed a total of 14 fragments of semideciduous Atlantic forest in Paraiba located in both rural (5 fragments) and urban areas (9 fragments, see Figure 2). The urban fragments consisted of two relatively large fragments (Mata do Buraquinho: 500 ha and Mata Timbo: 120 ha), and seven smaller forest fragments (size range: 2.8–8.7 ha, Table 1) located in the city of Joao Pessoa (>800,000 people) capital of Paraiba, northeastern Brazil (Figure 2). These urban fragments used to form a much larger continuous remnant of Atlantic forest until the 1960s, but are now separated by roads and buildings. We surveyed five rural forest fragments, which included: Gargau (1,058.62 ha) a privately owned conservation area located about 18 km north of the Mata do Buraquinho; Mata Pacatuba (266 ha) also a privately owned conservation area, located about 35 km west of Joao Pessoa city; Mata Asplan (96 ha), located 70 km north of Joao Pessoa; Mata Rio Tinto (339 ha, a protected area), 42 km north of Joao Pessoa; and Reserva Biologica Guaribas (REBIO, with 3,016 ha) located 52 km north of Joao Pessoa (Figure 2) where we sampled the well conserved forested area of Cabeca de Boi. All the fragments had experienced some degree of past logging.

**Table 1:** Fragments size, location, and sampling effort

Fragment	Survey date	Location	Area (ha)	# Transects	Sampling effort (km)
Mata Timbo (TI)	Feb–July 2015 and 2016	Urban, Joao Pessoa	120	3	4.2
Bioter	Feb–July 2015 and 2016	Urban, Joao Pessoa, Campus UFPB	7.5	2	6.6
Capel	Feb–July 2015 and 2016	Urban, Joao Pessoa, Campus UFPB	4.1	1	2.7
Biblio	Feb–July 2015 and 2016	Urban, Joao Pessoa, Campus UFPB	8.5	2	6.1
Reitoria	Feb–July 2015 and 2016	Urban, Joao Pessoa, Campus UFPB	8.7	2	4.6
HU	Feb–July 2015 and 2016	Urban, Joao Pessoa, Campus UFPB	2.8	1	2.9
Odonto	Feb–July 2015 and 2016	Urban, Joao Pessoa, Campus UFPB	3.8	2	4.6
LTF	Feb–July 2015 and 2016	Urban, Joao Pessoa, Campus UFPB	3.9	1	3.3
Mata Buraquinho (MB)	Feb–Dec 2015	Urban, Joao Pessoa	515	9	56.25^a^
Mata Gargau (GA)	Apr–Jun 2018	Rural, Santa Rita	1,056	13	34.02
Pacatuba^b^	Mar–Jun 2014	Rural, Sape	266	4	17.6
Mata Asplan^b^	Mar–Jul 2014	Rural, Mataraca	96.6	4	10.2
Rio Tinto^b^	Mar–Jun 2012	Rural, Rio Tinto	339	5	24
Cabeca de Boi, REBIO	Apr 2018	Rural, Mamamguape	3,000	7	2.1

a This sampling effort corresponds to the period when butterflies were sighted (Apr–Jun).

b Sampling effort in these areas was higher than those showed, giving the amount of time spent carrying out other studies.

Annual rainfall in the littoral area is around 1,500–1,700 mm and the average temperature is 25°C (Lima & Heckendorff, 1985). Floristic composition among the fragments is similar, but in the fragments of UFPB (Universidade Federal da Paraiba) there is a predominance of pioneer tree species (Barbosa, 1996).

### 2.2 Data collection butterflies

To collect data on ghost butterfly abundance, we used transect based distance sampling, which can provide accurate and unbiased estimates of population size and has a series of advantages (e.g., it is inexpensive, efficient, and allows robust modeling of population densities) in relation to other methodologies usually employed to estimate butterfly abundance, such as mark‐recapture or Pollard walk (Brown & Boyce, 1998; Isaac et al., 2011; Kral, Harmon, Limb, & Hovick, 2018).

We surveyed a total of 56 transects (range 1–13 per survey location, Table 1) that were walked between 07:00 and 16:00, to coincide with the daily activity period of the butterflies. Each transect was walked at a speed of about 1 km/hr and when a ghost butterfly was detected the sight distance, angle, and height were recorded. In the Gargau, the surveys were carried from April to June 2018 and REBIO Guaribas only in April 2018 (Table 1). The only fragments surveyed for two consecutive years, February to July 2015 and 2016, were those in the Campus of Universidade Federal da Paraiba (UFPB) and the Timbo fragment. We also recorded the presence of the common blue butterfly (*Morpho helenor*) in the fragments.

About four decades ago Kesselring and Ebert (1979) reported the presence of ghost butterflies in the MB fragment and noticed the seasonality of their appearance, recording their flight from mid‐April to the end of May. Strong seasonality also seems to be the norm in a closely related species, *M. epistrophus epistrophus*, that was reported to appear in March by Neves (2015), during a six‐month study (Oct–Mar) in a large Atlantic forest fragment (2,419 ha) in South Bahia. While Seitz (1924, cited in Young & Muyshondt, 1972) records this species flying in Rio de Janeiro from January to March.

We used a laser rangefinder to record distance to butterfly sightings. If a butterfly was stationary or resting, the distance was taken to its position or its position prior to an evasive movement. For butterflies in flight, distance was measured to the location where the butterfly was first noted. The ghost butterflies were easily spotted and differentiated from other species because of their size and color, they were usually found at short distance from the transects and their flight is slow, which are beneficial to facilitate the collection of data and reduce errors in measurements (Brown & Boyce, 1998).

### 2.3 Data on pollution levels – PM_2.5_

Many of the rural fragments were near sugarcane agriculture activities. During the sugarcane harvest, the crops are burned to facilitate the process of manual harvesting, which is demonstrated to generate high concentrations of air pollutants (Hall et al., 2012). We obtained air pollution estimates for particulate matter 2.5 μm or less in aerodynamic diameter (PM_2.5_) from the CATT‐BRAMS model (SISAM, 2018) which includes a PM_2.5_ tracer for biomass burning (Freitas et al., 2009). The CATT‐BRAMS model is a coupled chemistry aerosol‐tracer transport model developed for Brazil, which identifies biomass burning from remote sensing fire products and the mass of the emitted pollutant is estimated from field observations of vegetation burning (Longo et al., 2010). Air pollution estimates for each sampling location were obtained for the municipality which contains the sampling location. Fragments MB, UFPB, and Timbo were obtained from air pollution data for the Municipality of Joao Pessoa, and the Gargau fragment from the Municipality of Santa Rita. PM_2.5_ is well documented to have adverse health effects (Rückerl, Schneider, Breitner, Cyrys, & Peters, 2011). These data should be considered as relative concentrations because calibration sites are not available within the region. The preharvesting burning of the sugarcane in the areas we surveyed begins in July/August (Pereira & Silva, 2016; A. Campos personal communication to ACA).

### 2.4 Data analyses

We used distance 7.2 to obtain density estimates (Thomas et al., 2010) and corresponding coefficients of variation. We followed the recommendations of Thomas et al. (2010) and to model the detection functions we used half‐normal function with hermite polynomial expansion, uniform with cosine expansion and hazard‐rate with cosine expansion. The distance sampling analyses fit a detection function to the observed distance distribution, and we used this fitted function to estimate the proportion of individuals in the area (see Thomas et al., 2010). For the density estimation, we only consider the months that the butterflies were observed.

In our data, there were a spiking of observations near the transect line, most likely caused by rounding bearings close to the transect line to zero due to the butterfly's movements. We dealt with this in our analyses by binning the data into distance intervals to improve estimates of density and abundance (Buckland, Anderson, Burnham, & Laake, 2001). Since the ghost butterflies were found in only one fragment no comparison analyses were carried out.

## 3 RESULTS

### 3.1 Butterfly occurrence and density

**Figure 3:**
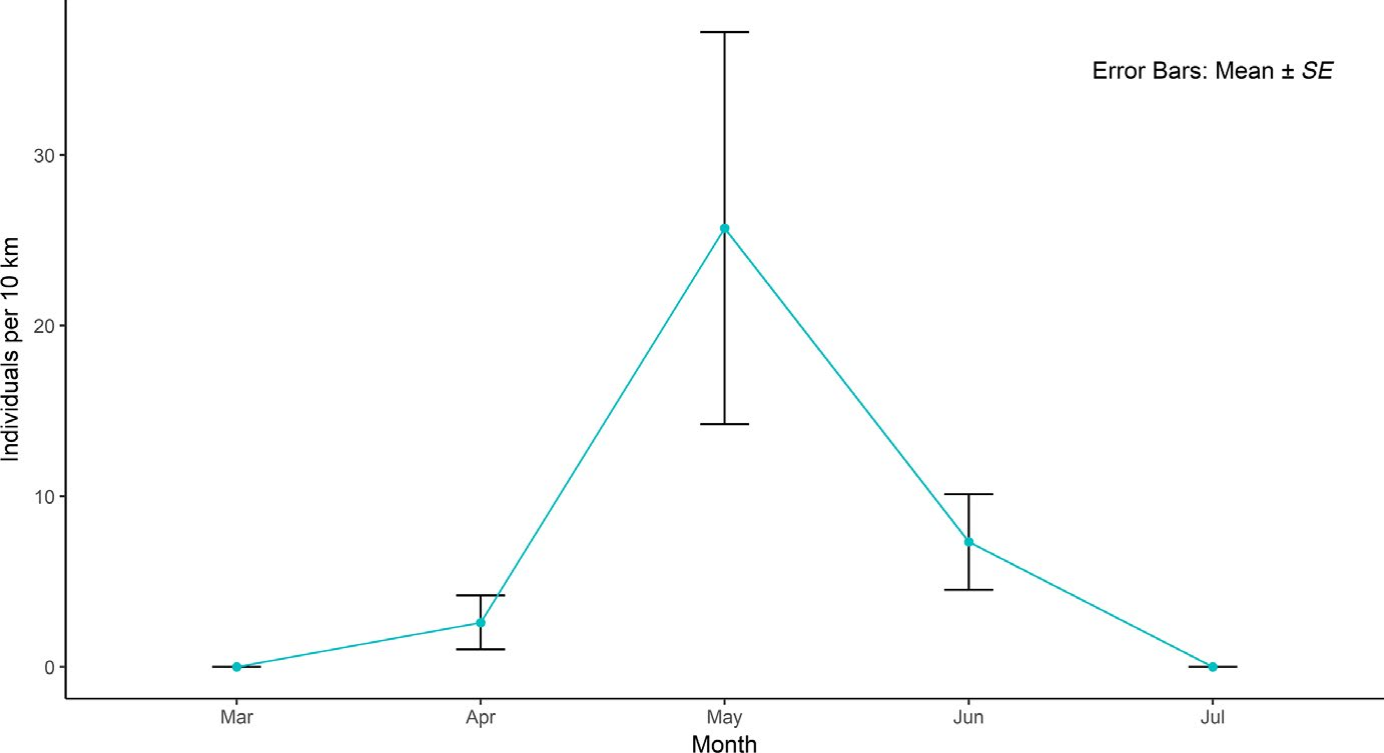
Seasonality in the appearance of the ghost butterfly in the Mata do Buraquinho urban fragment

We recorded a total of 99 ghost butterflies, all of which were observed in the Mata do Buraquinho (MB hereafter) urban fragment. The butterflies were strongly seasonal; the first individual was observed at beginning of April and by end of June no individual was observed (Figure 3). During the survey, the maximum number of butterflies observed in one transect was eight, but commonly the number of individuals we observed in the transects was lower (mean = 2.25 ± 1.86 *SD*, median = 1.5; mode = 1). The butterflies usually flew at heights between 1 m and 14 m (mean = 6.3 ± 2.8 *SD*, median = 6, mode = 6, *N* = 91) and most of time they seemed to be patrolling the transect. We also noticed the presence of the common blue butterfly (*M. helenor*, blue butterfly hereafter) in all the areas.

The model with the best fit for our data was the half normal with cosine adjustment and the estimate of density for MB was 1.4 ind/ha (95% CI 0.8–2.4 ind/ha) and we therefore estimate a total of 720 individuals (95% CI 425–1,220 ind) in the MB urban fragment. The relatively high variation in these estimates (CV = 24.1%) is explained by differences in sighting in the transects. These butterflies were sighted only inside the forest. They were never observed in open space or close to forest edge (<50 m). One of the transects run along a strip of forest (about 10–30 m wide and 200 m length) connecting forested areas but we never saw ghost butterflies there. In two transects we did not see ghost butterflies, although we saw them when returning and in other occasions when not surveying.

### 3.2 Pollution levels

**Figure 4:**
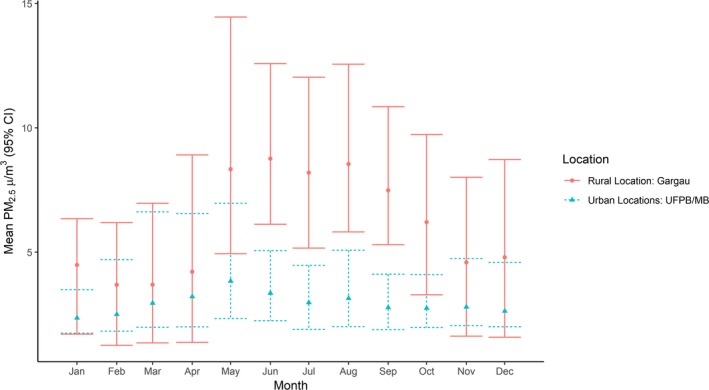
Monthly Mean PM2.5 Concentrations between 2007 and 2015 (excluding 2011), 95% confidence intervals are included

**Figure 5:**
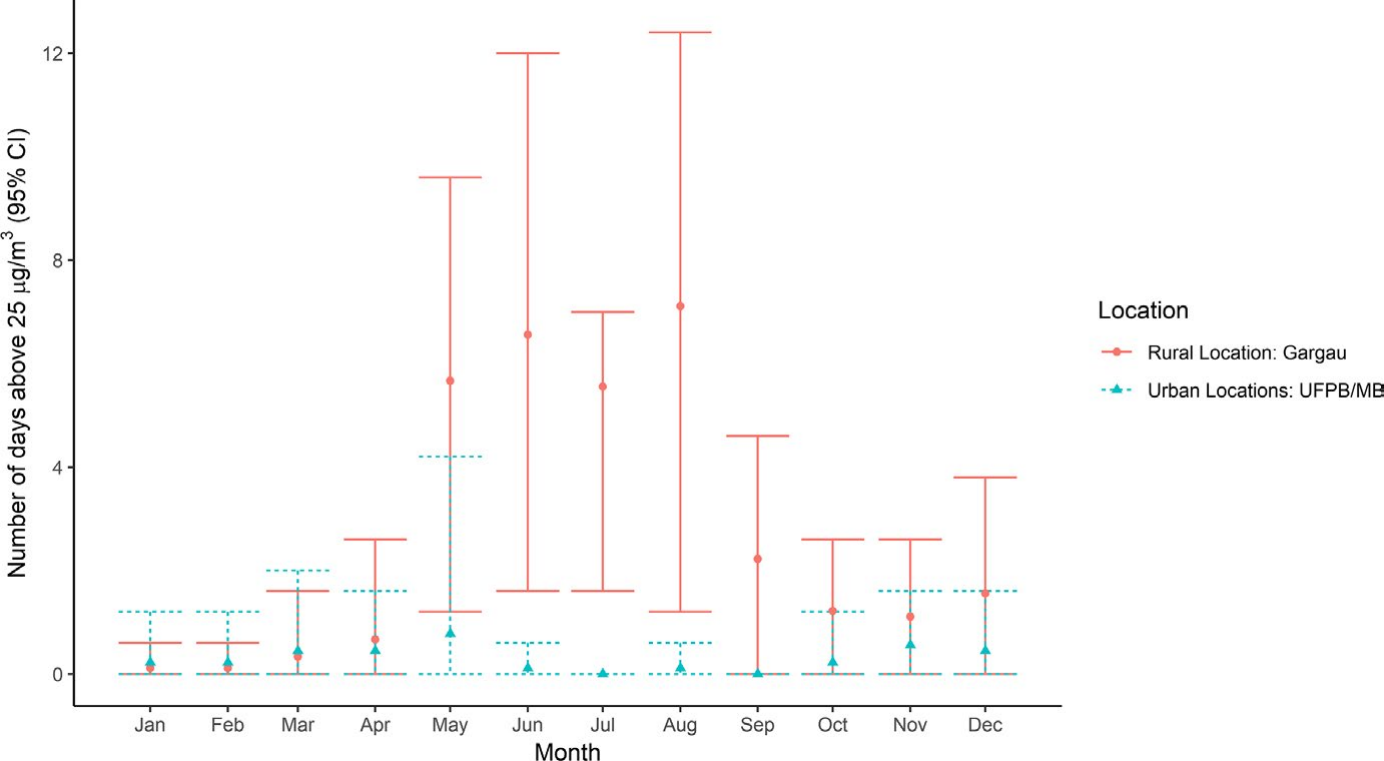
Monthly average number of days with concentrations exceeding 25 μg/m^3^ between 2007 and 2015 (excluding 2011), 95% confidence intervals are included

The Gargau fragment (rural location, see fragment labelled GA in Figure 2) had significantly higher levels of PM_2.5_ than Joao Pessoa (Figure 4). The air pollution estimates indicate a significant increase in the number of days per month where daily average air pollution concentrations are above 25 μg/m^3^ during the harvest season in the SR‐Gargau rural area (Figures 4 and 5) compared to urban sites (UFPB/MB).

## 4 DISCUSSION

Contrary to our hypothesis the ghost butterfly was absent from seemingly adequate rural forest fragments, for example, the larger Gargau forest fragment (>1,000 ha), yet they did occur in the smaller MB urban fragment (515 ha). Although we surveyed fragments that were near MB, such as the UFPB's and Timbo fragments (see Figure 2), the ghost butterfly did not occur in these smaller fragments. We did not find the ghost butterfly in the largest fragment (REBIO, 3,000 ha) that was surveyed for just one month, but long term and detailed studies also failed to report this species in the area (Villar, 2015). The blue butterfly, a similar‐sized species, was able to maintain their population in the small fragments. It was not uncommon to see individuals of this species moving between fragments of up to 30 m apart in UFPB and it occurred in all surveyed areas. The absence of the ghost butterfly from small urban fragments, near the larger MB, is interesting and indicates the fragility of this species to fragmentation and its inability to cross a more open matrix. Indeed, in MB we never observed the ghost butterfly flying in open space, not even in areas with sparse trees. It is possible that less vegetated areas could make it easier for predators to catch them and their absence from small urban fragments could be a consequence of high levels of predation by birds (Pinheiro & Cintra, 2017) causing the collapse of any remaining populations after the initial forest fragmentation. Indeed, *Galbula ruficauda*, a specialized butterfly predator, and several tyrant‐flycatchers are common in the UFPB campus, which prey upon on butterflies (Enedino, Loures‐Ribeiro, & , Santos, 2018; Pinheiro & Cintra, 2017). The removal of individuals by predators could create a top‐down regulatory mechanism that might explain our results for small fragments, but this consumptive effect on population abundance does not explain the ghost butterfly absence from larger nonurban fragments.

Some of the nonurban areas have similar size or are bigger than the urban MB fragment and were relatively close to MB, such as the Gargau forest fragment (over 1,000 ha, the second largest forest fragment in the region). Yet, the ghost butterfly does not occur in these fragments. One possible explanation for the puzzling absence of the ghost butterfly could be related to an inadequate amount of plant food resources for the butterflies (food resource for adults and larval host plants). The young caterpillars of the ghost butterfly show gregariousness (WM and ACA, personal observations), which has been linked to species inhabiting well conserved tropical wet forest (Young & Muyshondt, 1972). The closely related species (*Morpho epistophrus* and *Morpho catenarius*) also show cluster oviposition and strong larval gregariousness (Seitz, 1924 cited in Young & Muyshondt, 1972). Thus, rarity of plant resources could be reflected in local rarity or absence. Kesselring and Ebert (1979) recorded that caterpillars of the ghost butterfly feed on *Inga* spp., *Protium* spp. and other tree species. Two of these species (*Protium* spp and *Inga* sp) are among the most common trees, and saplings, in all the fragments (Barbosa, 1996, de Andrade, unpublished data). Therefore, unavailability of resources cannot be assumed. We cannot rule out the possibility that past disturbance and subtly differences in local climate might have had an influence in the ghost butterfly populations. For instance, the best‐conserved fragment is Pacatuba, but the average annual rainfall there is lower (<1,400 mm: Hue, Caubet, & Moura, 2017) than the other fragments, whereas the Gargau fragment has undergone a significant reduction in forest cover. It had a continuous forested area of over 5,000 ha in the 70's, but due to the sugarcane expansion about 80% of its areas was converted into plantations (Stevens, 2014). It is possible that years of logging or other disturbances in rural fragments could explain the ghost butterfly local extinction. Unfortunately, there is a dearth of information about the extent of past disturbance in the forest fragments we studied and how they might affect the *Morpho* butterflies. It is noteworthy; however, that the only population of the critically endangered *Morpho menelaus eberti* butterfly in Paraiba state occurs in the Gargau forest fragment (Melo, Filgueiras, Leal, & Freitas, 2014). Although there is some variability in the level of past anthropogenic disturbance (e.g., logging) across fragments, all of the rural fragments are immersed in a sugarcane matrix and for decades have been subjected to the stress (smoke pollution) from periodic fires; a common agricultural practice used in sugarcane plantation.

Our results showed that in the rural areas the levels of PM_2.5_ were significantly higher when compared to the urban area, and these high levels year‐round might be due to the sugarcane burning. The smoke and soot/ashes of the burning sugarcane are a known hazard for humans (Andrade, Cristale, Silva, Zocolo, & Marchi, 2010; Le Blond, Horwell, Williamson, & Oppenheimer, 2010; Mazzoli‐Rocha et al., 2014) and may have an impact on wildlife, but there is a dearth of studies and its effects are much less understood than, for instance, urban pollution (Lee, Davies, & Struebig, 2017; Isaksson, 2015; Mazzoli‐Rocha et al., 2014). The most toxic products of the sugarcane burning are aerosols (polycyclic aromatic hydrocarbons – PAHs) and small particulate matter (Godoi et al., 2004). Recently, Tan, Dion, and Monteiro (2018) evaluated, experimentally, the effects of smoke on the growth and survival of butterflies' caterpillar and found that smoke has detrimental effects on fitness. We suspect the byproducts of sugarcane burning might have a negative effect in the ghost butterfly population. Pesticide use could also be blamed (Kohler & Triebskorn, 2013), but the occurrence of the blue butterfly in nonurban fragments weakens this hypothesis. Interestingly, Uehara‐Prado, Brown, and Freitas (2007) recorded and captured a closely related species, *M. catenarius*, in larger (>10,000 ha) and small fragments (14–175 ha) of Atlantic forest in southeastern Brazil. Nevertheless, in this case the matrix surrounding the fragments were orchards, forestry and plantation of commercial timber, which is suggestive that the type of matrix and agricultural practices might impact these butterflies. Unfortunately, our data set does not allow the test of these possibilities. We urgently need further surveys in forest fragments not impacted by the sugarcane agricultural practice of burning to confirm if the ghost butterfly disappearance of Atlantic forest fragments could be due to byproducts of sugarcane burning, pesticides or due to past anthropogenic disturbance.

The ghost butterfly and closely related species (*M. epistophrus* and *M. catenarius*) take a long time to mature; adult individuals are found only once a year (Young & Muyshondt, 1972). Thus, they might be more sensitive to the byproducts of sugarcane burning and forest disturbance (see Ribeiro & Freitas, 2011), while the blue butterfly can be spotted throughout the year and probably has multiple generations within the year (Kesselring & Ebert, 1979). Probably, the impacts of sugarcane burning kept the ghost butterfly populations below a certain critical size, and below this critical size the populations were condemned to extinction; while blue butterfly that reproduce throughout the year, was able to maintain their population despite the localized anthropogenic impacts. The short temporal windows when adult ghost butterfly appear and mate, its larval gregariousness and possible cluster oviposition, the absence from relatively small urban forest fragments and the occurrence of the similar‐sized blue butterfly in all surveyed areas, suggest that the ghost butterfly may be under a strong Allee effect (Courchamp, Clutton‐Brock, & Grenfell, 1999). The Allee effect is a density dependent phenomenon, where the individual component of fitness is linked to population density (Courchamp et al., 1999) and it has been frequently reported in Lepidoptera (Fauvergue, 2013). Our explanation is speculative, but we believe merits further investigation.

Our results showed that a forest fragment immersed in an urban matrix and facing a number of anthropogenic pressures (de Andrade et al., 2019) still holds highly significant numbers of an endangered butterfly, which highlight the importance of urban forest remnants for conservation. The Atlantic forest in NE Brazil is extremely fragmented and dominated by small, isolated fragments (usually < 50 ha) covered in secondary growth vegetation (Ribeiro, Metzger, Martensen, Ponzoni, & Hirota, 2009), which exacerbate the risk of extinction for the ghost butterfly. It is possible that relatively large forest remnant in cities could be sustaining the last surviving populations of this species. There are reports of the ghost butterfly occurring in forest fragments near urban areas in larger cities elsewhere, but further surveys are needed to confirm their occurrence in larger urban forest fragments (Melo, Duarte, Mielke, Robbins, & Freitas, 2019).

We emphasize, however, that our findings should be considered with prudence, since we found the ghost butterfly in just a single urban site and this may limit our interpretation of the drivers for their local extinction in other fragments. Nonetheless, our data show indications that fragment size (at least in urban areas) has an adverse impact on ghost butterfly population and it appears likely that rural practice (such as sugar cane preharvesting burning) and past disturbance might underlie the pattern of local extinction. Our study shows the need of further autoecological studies to understand the process causing rarity of this species.

## CONFLICT OF INTEREST

None declared.

## AUTHOR CONTRIBUTIONS

ACA Designed the study, collected data, analyzed the data and wrote the paper. WM Collected the data, helped in part of analyses and contributed with early drafts. MA Acquired data on pollution, analyzed data and wrote the paper.

## Data Availability

Dryad Provisional https://doi.org/10.5061/dryad.tr62rm1.
